# Role of job and personal resources in the appraisal of job demands as challenges and hindrances

**DOI:** 10.1371/journal.pone.0248148

**Published:** 2021-03-29

**Authors:** Zofia Mockałło, Maria Widerszal-Bazyl

**Affiliations:** Laboratory of Social Psychology, Ergonomics Department, Central Institute for Labour Protection–National Research Institute, Warsaw, Poland; Universitat de Valencia, SPAIN

## Abstract

Previous research has shown that challenge and hindrance job demands show different effects on employees’ wellbeing and performance. Moreover, it has been demonstrated that employees’ subjective appraisal of job demands as challenges and hindrances may vary: they can be appraised as challenges or hindrances or both. Subjective appraisal of job demands was found to be also related to employees’ wellbeing and productivity. However, little is known about predictors of the appraisals of job demands made by employees. The aim of the study was to identify predictors of such appraisals among job and individual resources. Cross-sectional research was carried out among 426 IT, healthcare and public transport employees. COPSOQ II scales were used to measure job demands (emotional, quantitative, cognitive demands, work pace and role conflicts) and job resources (influence at work, possibilities for development, vertical and horizontal trust), single questions were used to measure employees’ subjective appraisals of job demands as hindrances and challenges, and PCQ was used to measure psychological capital. Multiple hierarchical regression analyses showed that only horizontal trust predicted the appraisal of job demands as challenges, and vertical trust predicted the appraisal of job demands as hindrances among four analysed job resources. Individual resource–psychological capital–predicted only the appraisal of job demands as challenges. Control variables–occupation, age and job demands also played a significant role in predicting the appraisal of job demands. Implications and future directions are discussed.

## Introduction

In recent years, there has been a lively discussion on the need to distinguish between two types of demands that lead to different psychological consequences: challenge and hindrance job demands [1]. Such challenges are defined as "work-related demands or circumstances that, although potentially stressful, have associated potential gains for individuals” [1, p. 68]. Challenges include job overload, time pressure, or high responsibility levels [1]. Hindrances are defined as “work-related demands or circumstances that tend to constrain or interfere with an individual’s work achievement and that do not tend to be associated with potential gains for the individual” [1, p. 68] and include organisational politics, red tape, or concerns about job security [1].

Research conducted in this field has indicated that hindrance demands are related to lower levels of positive affect and work engagement [2], increased emotional exhaustion and reduced vigour [3], increased employee turnover and lower job satisfaction [1], reduced performance [4], lower organisational commitment, and greater intent to quit, employee turnover, and withdrawal behaviour [5].

Most of the research in this field also indicated positive relationships of challenge demands with positive affect and work engagement [2], vigour [3], job satisfaction [1], work motivation and effectiveness [4], organisational commitment, as well as a negative relationship with the intent to quit and employee turnover [5]. However, some studies have also revealed that the challenge demands may be negatively related to employee wellbeing [6, 7].

As indicated by previous authors [8–11], subjective appraisal of demands as challenges or hindrances, in line with the transactional stress theory [12], is a significant factor explaining the discrepancies in the research results.

### Significance of subjective appraisal of demands as challenges or hindrances

Subsequent research has confirmed that cognitive appraisal of job demands as challenges or hindrances is separate from the challenge and hindrance demands themselves, as classified by researchers. It was proven that subjective appraisal of job demands was significantly related to employee wellbeing and productivity, coping strategies [10], strain and attitudes towards work [11]. It has also been found that the same job demands can be assessed as both a challenge and a hindrance [10, 11]. Finally, it was demonstrated that the appraisal of job demands as challenging and hindering might vary from day to day [11]. This line of research indicated that subjective appraisal partly explained differences in relationships between challenge and hindrance demands and employees’ wellbeing. However, little is known on the question of when and why employees assess demands as challenges and hindrances. Therefore, this study aimed to identify the predictors of job demands’ subjective appraisal as challenges and hindrances.

### Resources and subjective appraisal of job demands as challenges and hindrances

Preliminary answers to the question about the determinants of subjective appraisal of demands at work can be found in the Lazarus and Folkman’s transactional stress theory [12]. According to this theory, the threat appraisal occurs when the situation is goal-relevant, and the environmental demands significantly reduce or exceed the coping ability. In contrast, the challenge appraisal results from perceiving a situation as goal-relevant and the demands as not exceeding the coping ability and the available resources [13]. Thus, the available resources—social, psychological, physical, material [12–14] are relevant in the context of cognitive appraisal of demands. Therefore, we believe that also in a work situation, the determinants of challenge and hindrance appraisal should be sought in the individual resources of the employee and the resources available at work.

We draw from two lines of research: research on job resources included in the JDCS model [15] and JD-R model [16] as well as from related research on trust and social capital [17, 18] and underlying Conservation of Resources Theory [19].

That is why we decided to include influence at work, possibilities for development and horizontal and vertical trust as psychosocial job resources. In previous studies, job resources have been analysed as predictors of employee wellbeing and job attitudes, or as moderators or mediators of the relationships between work demands and employee wellbeing and job attitudes [16, 20–23]. However, job resources were not analysed as predictors of subjective appraisal of job demands.

Job control has been broadly tested concerning employees’ wellbeing. Job control in Karasek et al [24] JCQ is measured on two scales: decision authority and skill discretion, which seem semantically similar to COPSOQ II [25] scales of influence and possibility for development. Influence at work was proved to be a significant predictor of various wellbeing indicators, e.g. confidence and mental health [26], lower levels of burnout [27, 28], stress [28], higher job satisfaction [29], lower musculoskeletal pain [30], lower risk of long-term sickness absence [31], disability pensioning [32] or voluntary early retirement [33], higher levels of work ability and job engagement [34]. The level of control in the context of cognitive appraisal has been studied in terms of control over the stressor [35]; however, not over the work itself. Job control, influence at work is a key resource included in stress models [15, 16] that allows for coping with excessive work demands. Therefore, we believe that the sense of influence at work will increase the challenge appraisal and decrease the hindrance appraisal.

Possibility for development is a factor that describes the opportunity to develop and use skills [25]. Previously, it was demonstrated that possibility of development was related e.g. to self-rated health, burnout [28, 36, 37], job satisfaction [34, 38], mental health and perceived stress [37]. The definition of the challenge job demands refers precisely to the demands that, although they may be stressful, result in rewards such as an opportunity to learn or achieving desired goals [1]. Hence, we believe that the perceived overall possibilities for development at work will be positively associated with the challenge appraisal and negatively associated with the hindrance appraisal of job demands.

Trust is a predictor of employees’ attitudes and workplace behaviours [39], employees’ wellbeing [28, 40, 41], or organisational performance [40, 42, 43]. Trust is a key component of social capital [18, 44], which plays an important role in the cognitive appraisal [45], as well as in the relationship between the appraisal and its consequences [46]. It seems to be of especial importance when analysing hindrance and challenge appraisals at work, as according to Fukuyama [17] high level of trust allows for creating more flexible and group-focused working environments, where more responsibilities are delegated to the lower levels in organisational hierarchy. Low level of trust in the workplace is a factor isolating employees in frames of bureaucratic rules [17]. Therefore, it seems that low levels of trust are related to hindrance-appraisal, and high levels of trust are related to challenge-appraisals. More specifically, vertical trust describes the trust in management, supervisors [44, 47] due to reliability of the information they share with employees, their trust in employees and the fact that they allow employees to express their views and feelings [44]. Horizontal trust concerns mutual trust between employees in their daily work [44]. Therefore, we believe that both types of trust, as psychosocial job resources, will be important for the cognitive appraisal of job demands, where the relationships with both the management and colleagues are important.

As an individual resource, psychological capital consisting of optimism, hope, self-efficacy and resilience [48] was included in the present study. Research so far has shown significant relationships between psychological capital and employee performance, intent to quit, work happiness and subjective wellbeing [49], job satisfaction, organisational commitment, organisational citizenship behaviour [50]. Hence, this resource should support employees in a more positive appraisal of job demands: as something they can meet and subsequently, achieve their goals. This hypothesis is supported by previous research, e.g. demonstrating the role of self-efficacy [51] or psychological hardiness [52] in a cognitive appraisal.

In summary, four research hypotheses were formulated. Two of them concern the relationship between psychosocial work resources and employee appraisal of job demands as challenges and hindrances (H1a and H1b), and the remaining two concern the relationship between individual employee resource, i.e. psychological capital and employee appraisal of job demands as challenges and hindrances (H2a and H2b).

H1a: Influence, possibilities for development, horizontal trust, vertical trust, are positively related to the appraisal of job demands as challenges
H1b: Influence, possibilities for development, horizontal trust, vertical trust, are negatively related to the appraisal of job demands as hindrances
H2a: Psychological capital is positively related to the appraisal of job demands as challenges
H2b: Psychological capital is negatively related to the appraisal of job demands as hindrances

## Method

### Sample and procedure

The study group consisted of 426 participants, 56.9% of whom were men and 43.1% women. The mean age was 39.55 years (*Min* = 20; *Max* = 62), the mean job tenure was 14.53 years (*Min* = 1; *Max* = 42). Most of the participants had a higher education degree (52.4%) and the lowest number characterised participants with a post-secondary education degree (5.2%). The survey was conducted amongst health care nurses (150 participants), public transport employees (135 participants), IT employees (141 participants). Proportions of males and females were different in the three occupational groups, where most female workers participated in the health care nurses group (135 women), and most male workers participated in the IT (115 men) and public transport groups (112 men).

52% of the study group worked in the public sector, while 47.7% worked in the private sector. The largest number of participants held an employment contract of unlimited duration (82.2% of the group), and the lowest number held a contract for a specific task (0.2% of the group).

The cross-sectional questionnaire study was conducted through the external research agency in 41 organisations operating in 11 locations. On average, 5–10 employees from one organisation took part in the study. Consecutive sampling was applied. Interviewers contacted public and private sector organisations employing three chosen occupational groups asking for permission to conduct a questionnaire survey among their employees. After obtaining the permission, interviewers invited employees meeting study criteria according to age, gender, and occupation to participate in the study. Interviewers were presented information about the study, its anonymity, voluntary participation, ability to resign at any point. Participants willing to take part, received a set of questionnaires to be filled in using the paper-pencil method. The survey was anonymous. The questionnaires were completed by the respondents at their convenience. Next, the interviewer was responsible for collecting them on a pre-defined date. The respondents sealed the completed questionnaires in envelopes provided in advance in order to ensure confidentiality and prevent unauthorised access by other persons (e.g. superiors).

### Ethics

The research was conducted according to the Polish National Academy of Sciences code of ethics [53] and the European Commission principles on ethics in social science and humanities [54]. Participants were informed that their participation in the study was voluntary and they were free to stop participation at any point. They were aware that the questionnaire study was anonymous, and no personal information was gathered. Study participants were also informed that data collected from them would be included in further statistical analysis.

Additionally, we followed Whicher and Wu [55] consideration for determining the necessity of ethics oversight for survey research. According to this, because study participants were not vulnerable subjects, there was no risk of informational or psychological harm, no institutional ethics oversight was necessary.

The Polish Bioethical Commission is responsible for supervising clinical trials and medical research. Questionnaire research, surveys on psychosocial working conditions and wellbeing are not a medical experiment; thus there are no relevant regulations regarding such surveys in Poland. CIOP-PIB does not have its own IRB.

### Measures

Cronbach alpha values of the measures used in the study are presented in Table 1. Most of the scales are characterised with good reliability [56], while vertical trust and role conflict scales’ Cronbach alpha values are slightly under 0.7 (0.68 and 0.67, respectively), which is still acceptable in case of scales consisting of a low number of items [57].

**Table 1:** Descriptive statistics and correlations for key study variables (N = 426).

	*α*	*M*	*SD*	*Min*	*Max*	1	2	3	4	5	6	7	8	9	10	11	12	13
1. Age		39.67	10.19	20.00	62.00	-												
2. Emotional demands	0.87	57.44	22.44	0.00	100.00	0.27***	-											
3. Cognitive demands	0.82	71.28	17.99	12.50	100.00	-0.002	0.33***	-										
4. Quantitative demands	0.73	39.19	17.25	0.00	81.25	0.01	0.06	0.06	-									
5. Work pace	0.84	60.38	21.16	0.00	100.00	0.05	0.38***	0.48***	0.15**	-								
6. Role conflicts	0.67	33.52	20.60	0.00	100.00	0.11*	0.31***	0.18***	0.32***	0.21***	-							
7. Influence	0.73	48.96	21.38	0.00	100.00	0.004	0.11*	0.38***	0.14**	0.19***	0.06	-						
8. Possibilities for development	0.81	65.57	18.91	6.25	100.00	-0.15**	0.18***	0.45***	-0.04	0.35***	-0.05	0.44***	-					
9. Horizontal trust	0.71	64.22	19.09	0.00	100.00	-0.11*	-0.05	0.07	-0.10*	0.04	-0.39***	-0.5	0.25***	-				
10. Vertical trust	0.68	61.38	16.10	12.50	100.00	-0.05	0.05	0.11*	-0.16**	0.09	-0.32***	0.06	0.36***	0.60***	-			
11. Psychological capital	0.92	100.18	16.94	27.73	144.00	-0.21***	0.02	0.24***	-0.22***	0.15**	-0.21***	0.27***	0.40***	0.40***	0.29***	-		
12. Global appraisal of job demands as challenges	0.85	4.74	1.08	1.00	7.00	-0.05	0.03	0.28***	-0.07	0.22***	-0.03	0.12**	0.24***	0.29***	0.19***	0.32***	-	
13. Global appraisal of job demands as hindrances	0.88	4.12	1.16	1.00	7.00	0.12*	0.04	0.08	0.16**	0.16**	0.18***	-0.01	-0.08	-0.10*	-0.26***	-0.09	0.31***	-

*p < .05.

**p < .01.

***p < .001.

#### Level of job demands

The study participants assessed the level of five types of job demands using the Copenhagen Psychosocial Questionnaire [25] selected scales. The COPSOQ had already been adapted earlier to Polish conditions and used in numerous Polish studies. It refers to several COPSOQ I scales [34] and to COPSOQ II scales [58] used in the present study.

For the scales of cognitive, quantitative, emotional, and work pace demands, 5-point response scales were provided, where 1 meant “always”, 5 meant “never/ hardly ever”. The values obtained were then calculated according to the COPSOQ authors into 0 (for “Never/Hardly never” or “To a very small extent”), 25, 50, 75 and 100 points (for “Always” or “To a very large extent”) and the scale score was calculated as the mean item score.

*Cognitive demands*—measured using the 4-item COPSOQ II scale. Example question: “Does your work require that you remember a lot of things?”
*Quantitative demands*—measured using the 4-item COPSOQ II scale. Example question: “Is your workload unevenly distributed so it piles up?”
*Work pace (tempo)*—measured using the 3-item COPSOQ II scale. Example question: “Is it necessary to keep working at a high pace?”
*Emotional demands*—measured using the 4-item COPSOQ II scale. Example question: “Do you get emotionally involved in your work?”
*Role conflicts*—measured using the 4-item COPSOQ II scale. In the case of this scale, also 5-point Likert scales were presented, but 1 meant “to a very large extent", 5 “to a very small extent”. An example question: “Do you do things at work which are accepted by some people but not by others?”

#### Subjective appraisal of job demands as challenges and hindrances

The appraisal of demands as challenges and hindrances was carried out using the modified method presented by Webster, Beehr and Love [11]. After assessing the level of five types of demands (see above), the respondents were asked to familiarise themselves with the definitions of challenge and hindrance job demands according to the definition introduced by Cavanaugh et al. [1], and then, on the basis of these definitions, they were asked to assess the different types of demands on 7-point challenge scales and 7-point hindrance scales, where 1 meant “strongly disagree”, 7 –“strongly agree”. For example, after determining the level of cognitive demands using the COPSOQ II questionnaire scale, the respondents were asked to appraise the extent to which the cognitive demands they experienced in their work were a challenge (on a scale from 1 to 7) and a hindrance (on a scale from 1 to 7). As in the study by Webster, Beehr and Love [11], each factor could be assessed in two ways (hindrance and challenge). Next, the appraisals of each demand on the scales of challenges and hindrances were averaged into two indicators: Global appraisal of job demands as challenges (mean of appraisals of five types of job demands as challenges) and Global appraisal of job demands as hindrances (mean of appraisals of five types of job demands as hindrances).

#### Psychosocial job resources

Psychosocial job resources, i.e. influence, possibilities for development, horizontal trust, vertical trust were measured using the COPSOQ II scales (Copenhagen Psychosocial Questionnaire) [25]. The respondents were asked to assess the level of available resources by marking an appropriate number on the 5-point Likert scale, where 1 = “Always” or “To a very large extent”, and 5 = “Never / hardly ever” or “To a very small extent”. As described above, the values obtained were then calculated according to the COPSOQ authors into 0 (for “Never/Hardly never” or “To a very small extent”), 25, 50, 75 and 100 points (for “Always” or “To a very large extent”) and the scale score was calculated as the mean item score.

*Influence*—was measured using a 4-item scale. Example question: “Do you have a large degree of influence concerning your work?”
*Possibilities for development*—were measured using a 4-item scale. Example question: “Can you use your skills or expertise in your work?”
*Horizontal trust–*was measured using a 3-item scale. Example question: “Do the employees withhold information from each other?”
*Vertical trust*—was measured using a 4-item scale. Example question: “Can you trust the information that comes from the management?”

#### Psychological capital

Psychological capital was measured using the Psychological Capital Questionnaire [59], which contains 24 questions forming four sub-scales: Optimism, Hope, Self-efficacy and Resilience. Respondents are asked to mark appropriate value on the scale ranging from 1 to 6 in order to indicate to what extent they agree with the statements. Sample items: “If I should find myself in a jam at work, I could think of many ways to get out of it”, “I usually take stressful things at work in stride”.

### Statistical analysis

The analyses were performed using the SPSS software version 21. The Markov Chain Monte Carlo (MCMC) multiple imputation of missing value technique was used to deal with missing data in the database, simulating 5 complete datasets using 100 iterations. Missing values were observed in case of most variables analysed, apart from emotional demands level. The highest number of missing values was observed in appraisal of cognitive demands as hindrances (25 missing values and 125 imputations), and the lowest number of missing values was observed in case of cognitive demands level, quantitative demands, time pressure, influence (2 missing values and 10 imputations in each case).

Preliminary analyses included a correlation analysis. Student’s t-test and one-factor ANOVA were performed to identify any significant differences in the appraisal of job demands as challenges and hindrances between genders and occupational groups. Additionally, the Student’s t-test and one-factor ANOVA were performed not only for the global appraisal of job demands as challenges and hindrances but also for the challenge and hindrance appraisals made for each type of job demand separately. This analysis aimed to gain more insight into the final, global appraisal indicators and their nature.

In order to analyse the relationship between job resources and individual resources and the global appraisal of job demands as challenges and hindrances, two hierarchical multiple regression analyses were conducted. In the first analysis, the dependent variable was the global appraisal of job demands as challenges. This indicator was created as an average appraisal of job demands (i.e. cognitive, emotional, quantitative demands, tempo and role conflicts) as challenges. In the second regression analysis, the dependent variable was the global appraisal of job demands as hindrances. This indicator was created as an average appraisal of job demands as hindrances.

Step 1 and Step 2 contained the control variables (Step 1 –age, occupational group; Step 2 –job demands). The predictor variables were entered in Steps 3 and 4 (Step 3 –psychosocial job resources; Step 4 –psychological capital).

The Durbin-Watson test was performed in order to check if data in tested models were auto-correlated, and Variance Inflation Factors (VIF) test was also performed, excluding the possibility of multicollinearity in the regression analysis. Because the vertical and horizontal trust scales were highly correlated, separate models were also tested to confirm that multicollinearity regarding the two dimensions of trust was not an issue.

## Results

### Correlations analysis

Descriptive statistics and correlation coefficients of the analysed variables are presented in Table 1.

The global appraisal of job demands as challenges correlated positively with all the available resources, both concerning psychosocial job resources and individual resources in the form of psychological capital. The global appraisal of job demands as hindrances correlated with horizontal and vertical trust (negatively) and with psychological capital (contrary to our anticipations, positively). The results also revealed that the global appraisals of job demands as challenges and as hindrances were positively correlated with each other.

Additionally, the correlation analysis demonstrated that the global appraisal of job demands as challenges was positively related with two types of job demands, i.e. cognitive demands and tempo (work pace). In contrast, the global appraisal of job demands as hindrances was positively related to age, and three types of job demands, i.e. quantitative demands, tempo, and role conflicts. Moreover, emotional demands were found not to be correlated with the global appraisal of job demands as challenges or hindrances. We will proceed to the interpretation of this result after reporting the results of the remaining analyses.

#### Appraisal of job demands as challenges and hindrances in groups differentiated by gender and occupation

A one-factor ANOVA analysis of variance showed that there were significant differences in groups differentiated by occupation (IT, public transport, health care) in the average level of appraisal of job demands as challenges, while the average level of appraisal of job demands as hindrances remained equal (did not differ significantly). The results of the analysis are presented in Table 2.

**Table 2:** Results of ANOVA, NIR and Games-Howell post-hoc tests for subjective appraisal of job demands as hindrances and challenges in three occupational groups.

Variable	Group						
	IT N = 141		Public transport N = 135		Healthcare N = 150		
	*M*	*SD*	*M*	*SD*	*M*	*SD*	*F*
Challenge appraisal of emotional demands	5.18^a^^.^^c^	1.17	4.63	1.78	4.70	1.51	6.94**
Hindrance appraisal of emotional demands	4.14	1.40	4.09	1.53	4.08	1.59	0.12
Challenge appraisal of cognitive demands	5.56^a^^.^^c^	0.94	4.58	1.38	4.67	1.41	33.28***
Hindrance appraisal of cognitive demands	3.81	1.45	3.99	1.57	4.08	1.49	1.23
Challenge appraisal of quantitative demands	5.16^a^^.^^c^	1.22	4.28	1.51	4.46	1.44	17.57***
Hindrance appraisal of quantitative demands	4.28	1.43	3.97	1.48	4.21	1.56	1.67
Challenge appraisal of work pace	5.32^a^^.^^c^	1.01	4.61	1.37	4.52	1.36	20.87***
Hindrance appraisal of work pace	4.13	1.42	4.20	1.33	4.19	1.43	0.12
Challenge appraisal of role conflicts	4.70^c^	135	4.43	1.30	4.35	1.31	2.60
Hindrance appraisal of role conflicts	4.26	1.40	4.18	1.27	4.18	1.32	0.15
Global appraisal of job demands as challenges	5.18^a^^.^^c^	0.88	4.50	1.10	4.54	1.12	22.33***
Global appraisal of job demands as hindrances	4.12	1.20	4.09	1.11	4.15	1.19	0.10

Results of NIR and Games-Howell post-hoc tests:

^a^ IT vs Public transport–*p*<0,05;

^b^ Public transport vs Healthcare–*p* < 0,05;

^c^ IT vs Healthcare–*p* < 0,05.

*p < .05.

**p < .01.

***p < .001.

Note: Since Levene’s test showed that the variance in occupational groups is not homogeneous in the case of the variables: “Challenge appraisal of emotional demands”, “Challenge appraisal of cognitive demands”, “Challenge appraisal of quantitative demands”, “Challenge appraisal of work pace”, and “Global appraisal of job demands as challenges”, for these variables *F* Welch statistics are presented.

Significant differences were observed in challenge appraisal levels between the IT employees, on the one hand, and the healthcare and public transport employees, on the other. The IT employees appraised job demands as challenges significantly higher than the employees in the other two groups: not only in terms of the global appraisal of job demands as challenges but also in relation to each type of job demands. In other words, the IT employees appraised emotional, cognitive, quantitative demands, as well as tempo and role conflicts as challenges significantly higher than the other two groups. There were no differences in the appraisal of demands as challenges between public transport and healthcare employees. There were also no differences in terms of evaluating job demands as hindrances–neither in terms of global appraisal of job demands, nor appraisal of each type of job demand.

In order to analyse gender differences in the appraisal of job demands as challenges and hindrances, a Student’s t-test was conducted. The Levene’s test proved homogeneity of variance between the groups for most variables, except for the appraisal of tempo (work pace) as a challenge. In the case of this variable, a Welch correction was applied.

The analysis conducted showed only one significant difference between both genders, in the work pace appraisal as a challenge: men appraised work pace as a challenge higher than women. There were no significant differences in terms of challenge and hindrance appraisal in the case of other job demands nor in the case of the global appraisal of job demands as challenges and hindrances (Table 3).

**Table 3:** Mean appraisals of job demands as challenges and hindrances and Student’s T-test results in women and men.

Variable	Group				
	Women N = 182		Men N = 240		
	*M*	*SD*	*M*	*SD*	*t*
Challenge appraisal of emotional demands	4.75	1.47	4.90	1.55	-0.95
Hindrance appraisal of emotional demands	4.07	1.48	4.13	1.53	-0.46
Challenge appraisal of cognitive demands	4.92	1.38	4.95	1.30	-0.23
Hindrance appraisal of cognitive demands	4.02	1.50	3.92	1.51	0.63
Challenge appraisal of quantitative demands	4.53	1.52	4.71	1.38	-1.24
Hindrance appraisal of quantitative demands	4.14	1.56	4.17	1.44	-0.18
Challenge appraisal of work pace	4.66	1.40	4.93	1.22	-2.06*
Hindrance appraisal of work pace	4.14	1.43	4.20	1.37	-0.42
Challenge appraisal of role conflicts	4.41	1.35	4.55	1.30	-1.10
Hindrance appraisal of role conflicts	4.24	1.31	4.18	1.35	0.43
Global appraisal of job demands as challenges	4.65	1.15	4.81	1.02	-1.45
Global appraisal of job demands as hindrances	4.12	1.15	4.12	1.18	-0.01

*p < .05.

**p < .01.

***p < .001.

Note: For the variable: „Challenge appraisal of work pace” *t* statistics with the correction for unequal variances are presented.

The results presented in Tables 2 and 3 also show that regardless of whether the study group was divided according to gender or occupation, all the subgroups showed similar regularities. Namely, each of the five types of job demands was appraised as both a challenge and a hindrance, and the appraisals of each type of job demands as a challenge were higher than as a hindrance.

Such dominance of challenge appraisals over hindrance appraisals seemed particularly pronounced in case of cognitive demands and emotional demands. In case of other types of job demands, in particular the role conflict, the dominance of challenge appraisals over hindrance appraisals was minimal.

### Psychosocial and individual resources and global appraisal of job demands as challenges

The regression analysis results of the global appraisal of job demands as challenges are presented in Table 4.

**Table 4:** Summary of hierarchical regression analysis for variables predicting global appraisal of job demands as challenges (*N* = 426).

	Model 1			Model 2			Model 3			Model 4		
Predictors	*B*	*SE B*	*Β*	*B*	*SE B*	*β*	*B*	*SE B*	*β*	*B*	*SE B*	*β*
Age	0.01	0.01	0.11*	0.01	0.01	0.10*	0.01	0.01	0.10	0.01	0.01	0.10*
Public transport	-0.81	0.14	-0.35***	-0.86	0.14	-0.37***	-0.69	0.15	-0.30***	-0.63	0.15	-0.27***
Healthcare	-0.73	0.13	-0.32***	-0.70	0.14	-0.31***	-0.65	0.15	-0.29***	-0.59	0.15	-0.26***
Emotional demands				0.01	0.01	0.05	0.01	0.01	0.03	0.01	0.01	0.02
Cognitive demands				0.01	0.01	0.17**	0.01	0.01	0.15**	0.01	0.01	0.15**
Quantitative demands				-0.01	0.01	-0.11*	-0.01	0.01	-0.10*	-0.01	0.01	-0.08
Work pace				0.01	0.01	0.16**	0.01	0.01	0.15**	0.01	0.01	0.14**
Role conflict				-0.01	0.01	-0.04	0.01	0.01	0.06	0.01	0.01	0.06
Influence							-0.01	0.01	-0.02	-0.01	0.01	-0.04
Possibilities for development							0.01	0.01	0.02	0.01	0.01	-0.01
Horizontal trust							0.01	0.01	0.21***	0.01	0.01	0.17**
Vertical trust							0.01	0.01	0.02	0.01	0.01	0.02
Psychological capital										0.01	0.01	0.14*
*R*^*2*^	0.09			0.18			0.21			0.22		
*F* for change in *R*^*2*^	14.41***			10.09***			5.22***			6.48*		

*p < .05.

**p < .01.

***p < .001.

Note: Categorical variable “Occupation” was coded as follows: 0 –IT, 1 –Public transport and healthcare.

Adjusted *R*^*2*^ values are presented.

The control variables introduced in step 1, i.e. age and occupational group, were found to be significant predictors–together, they explained 9% of the variance of the global appraisal of job demands as challenges. The regression coefficients of these variables obtained in the last regression step indicate that the older the employee, the more he/she assessed the demands at work as challenges, and that compared to the IT industry, the public transport employees and health care employees appraised job demands as challenges to a lesser extent.

The levels of job demands (i.e. emotional, cognitive, and quantitative demands, work pace and role conflicts) introduced in Step 2 explained in total 9% of the variance of global appraisal of demands as challenges. However, in the last step of the regression, only two of them proved to be significant predictors: cognitive demands and work pace). That is, the higher the level of cognitive demands and work pace, the higher the global appraisal of job demands as challenges.

Among the psychosocial job resources introduced in Step 3 that explained 3% of the variance in the level of appraisal of job demands as challenges (*F*(12,413) = 10.26; *p* < 0.001), only horizontal trust was a significant predictor. The higher the level of trust between employees, the higher the appraisal of job demands as challenges.

The introduction of psychological capital in Step 4 caused a 1% change in *R*^*2*^ (*F*(13,412) = 10.10; *p* < 0.05). The psychological capital was positively related to the global appraisal of work demands as challenges. In this step, the horizontal trust remained a significant predictor of the appraisal of job demands as challenges, and among the individual types of job demands, cognitive demands, and time pressure remained significant.

In other words, the hypothesis 1a was partially confirmed. Of the four types of psychosocial work resources, only one type, i.e. horizontal trust was significantly related to the level of global appraisal of job demands as challenges.

On the other hand, the hypothesis 2a was fully confirmed: psychological capital predicted the level of global appraisal of job demands as challenges, although the degree of the variance explained was very low.

#### Psychosocial job resources and individual resources and global appraisal of job demands as hindrances

The regression analysis results of the subjective appraisal of job demands as hindrances are presented in Table 5. Among the controlled variables introduced in Step 1, only age was a significant predictor of the global appraisal of job demands as hindrances: the older the employees were, the more they appraised demands as hindrances.

**Table 5:** Summary of hierarchical regression analysis for variables predicting global appraisal of job demands as hindrances (*N* = 426).

	Model 1			Model 2			Model 3			Model 4		
Predictors	*B*	*SE B*	*β*	*B*	*SE B*	*β*	*B*	*SE B*	*β*	*B*	*SE B*	*β*
Age	0.02	0.01	0.16**	0.02	0.01	0.15**	0.02	0.01	0.14**	0.02	0.01	0.14**
Public transport	-0.24	0.16	-0.09	-0.20	0.16	-0.08	-0.22	0.17	-0.09	-0.24	0.17	-0.10
Healthcare	-0.12	0.14	-0.05	-0.17	0.17	-0.07	-0.12	0.17	-0.05	-0.14	0.17	-0.06
Emotional demands				-0.01	0.01	-0.06	-0.01	0.01	-0.04	-0.01	0.01	-0.04
Cognitive demands				-0.01	0.01	-0.01	0.01	0.01	0.05	0.01	0.01	0.05
Quantitative demands				0.01	0.01	0.10	0.01	0.01	0.07	0.01	0.01	0.06
Work pace				0.01	0.01	0.14*	0.01	0.01	0.17**	0.01	0.01	0.17**
Role conflict				0.01	0.01	0.14**	0.01	0.01	0.07	0.01	0.01	0.07
Influence							0.01	0.01	0.01	0.01	0.01	0.01
Possibilities for development							-0.01	0.01	-0.08	-0.01	0.01	-0.07
Horizontal trust							0.01	0.01	0.10	0.01	0.01	0.12
Vertical trust							-0.02	0.01	-0.28***	-0.02	0.01	-0.28***
Psychological capital										-0.01	0.01	-0.04
*R*^*2*^	0.01			0.06			0.12			0.12		
*F* for change in *R*^*2*^	3.00*			5.44***			7.54***			0.62		

*p < .05.

**p < .01.

***p < .001.

Note: Categorical variable “Occupation” was coded as follows: 0 –IT, 1 –Public transport and healthcare.

Adjusted *R*^*2*^ values are presented.

Job demands introduced in Step 2 explained 5% of the variance of global appraisal of job demands as hindrances. While in Step 2, two job demands (work pace and role conflicts) were important predictors of the global appraisal, in the last step of the regression only work pace proved to be an important predictor of global appraisal of job demands as hindrances.

Among the psychosocial job resources introduced in Step 3, only vertical trust was a significant predictor of job demands’ global appraisal as hindrances. This model explained 6% variance in the dependent variable (*F*(12.413) = 5.76; *p* < 0.001). The greater the vertical trust, the lower was the employee global appraisal of job demands as hindrances.

Adding psychological capital to the model in Step 4 did not increase its predictive value, and the psychological capital itself appeared to be unrelated to the appraisal of job demands as hindrances (*F*(13.412) = 5.36; *p* < 0.001). Still, the only significant variable among job and individual resources was vertical trust.

Thus, hypothesis 1b was partly confirmed. Of the four types of psychosocial job resources, only vertical trust proved to be positively related to job demands’ subjective appraisal as hindrances.

However, the hypothesis 2b was not confirmed. It was found that psychological capital was not significantly related to the subjective appraisal of job demands as hindrances.

## Discussion

The study results confirmed that employee could assess the same job demands as both challenges and hindrances. The results obtained are in line with previous authors [10, 11, 60] and the transactional stress theory [12], which also assumed that an individual could simultaneously assess an event both as a challenge and a threat [11]. Moreover, it was confirmed that job and individual resources are related to the individual appraisal of job demands. Our results suggest that including this current of research could help gain a more thorough understanding of the role of job demands and resources play in employees’ working lives.

### Psychosocial job resources and appraisal of demands as challenges and hindrances

According to the hypothesis 1, it was assumed that the appraisal of job demands as challenges would be positively related to psychosocial job resources, and the appraisal of job demands as hindrances would be negatively related to the level of job resources. This hypothesis was partially confirmed: out of four psychosocial work resources included; two proved to be important predictors of job demands’ appraisal. Horizontal trust, i.e. trust in co-workers, was the predictor of appraisal of job demands as challenges, while vertical trust, i.e. trust in management, was the predictor of appraisal of job demands as hindrances.

In general, trust in the workplace’s social environment plays an important role in job demands’ appraisal can be relatively easily understood. Trust in others gives a sense of security [61] and is positively related with subjective wellbeing [62–65], as well as promotes interpersonal cooperation [66–68]. Positive affect, wellbeing or enhanced cooperation are probably related to the employees’ perception of the tasks as challenges they can cope with, and to a lesser extent as hindrances difficult to overcome.

The results indicating different roles of horizontal and vertical trust in the appraisal of demands as challenges and hindrances are more complex, further complicated by the fact that both types of trust were highly correlated. Other studies also showed strong relationships between the two types of trust [69]. In the light of our results, it should be considered that the two types of trust, despite their strong interrelationships, are functionally independent. In other words, they have different functions in relation to external events. This has already been demonstrated by other researchers studying the role of two from of trust in relation to organisational engagement and organisational citizenship behaviour [70], as well as knowledge sharing [71]. Coming back to our result, there are two possible explanations.

First, the result could be explained by the difference in psychological distance between the employee—colleague relationship and the employee–supervisor/management relationship. Many Polish organisations are managed in an authoritarian way [72]. Therefore, there is a considerable psychological distance between the employee and management. Friendly relations are characterised by a closer distance, they are often more frequent, more direct, and are part of employees’ everyday life. This is probably why trust in colleagues gives a sense of security on a daily basis, promotes cooperation, and determines a more optimistic perception of the tasks or demands employees face and treat as challenges.

Secondly, the above result may have been caused by the fact that it is primarily the manager who delegates tasks and sets the demands at work, not the colleagues. If the manager determines complex demands that are difficult to meet and appraised as hindrances, he or she will be treated as guilty of the situation. Therefore, it is likely that the employee expects the manager in the first place not to impose hindering demands that are impossible or difficult to meet. Moreover, trust in the management means that the employee does not see the demands as hindrances.

The remaining two psychosocial resources considered in this study, i.e. influence at work and possibilities for development, although correlated with the global appraisal of job demands as challenges, have not been found to be predictors of subjective appraisal of job demands as challenges or hindrances. In the case of possibilities for development, the close relationship between this job resource and psychological capital may offer an explanation. This may mean that employees with high psychological capital also perceive greater possibilities for development, and vice versa–the job resources in the form of high possibilities for development support the development of psychological capital of employees, as a result of which they are conducive to assessing the demands of work as challenges.

The level of influence at work, contrary to the hypotheses, was not significantly related to the appraisal of job demands. Although the correlation analysis showed a significant relationship between the level of influence and appraisal of job demands as challenges, the regression analysis, with other potential predictors included and controlled for, did not show a significant relationship between these factors. In the case of appraisal of job demands as hindrances, no significant relationships with the level of influence at work were noted in either correlation nor regression analysis. This result seems to be inconsistent with Peeters, Buunk and Schaufeli [73], where the ability to control stressful events at work was the most important variable in the cognitive appraisal. On the other hand, it should be noticed that we measured the overall level of influence at work, rather than specific forms of control over particular types of demands. For example, a specific form of control appropriate to emotional demands could be learnt control over personal emotions, and a form of control appropriate to cognitive demands would be intellectual control over the material to which the work relates, and so on. This idea refers to the DISC model by de Jonge and Dormann [74], which assumed that these types of resources reducing the negative impact of a stressor should be related to the type of stressor.

### Psychological capital and the appraisal of job demands as challenges and hindrances

Contrary to the hypothesis, psychological capital was not found to be significantly associated with the appraisal of job demands as hindrances. The relationship between psychological capital and the appraisal of job demands as challenges was strong in the correlation analysis, while in the regression analysis, with sociodemographic and psychosocial variables controlled for, it lost its strength.

The result showing the different role of psychological capital in relation to appraisal of job demands as challenges and hindrances resembles the results obtained by Min, Kim and Lee [75]. These authors found that psychological capital buffered the negative impact of challenge job demands on work engagement, but not the impact of hindrance job demands. In interpreting the results, authors refer to a general thesis upholding that in particularly difficult situations, individual differences between people do not play as much of a role as in less difficult situations. When an individual is confronted with hindrance job demands, even a high level of psychological capital is not a sufficient resource to cope with such a situation, leading to loss of work engagement, presented by Min, Kim, and Lee [75], nor does it allow for a more optimistic appraisal of job demands—they are perceived as hindrances no less than by employees with a low level of psychological capital, as shown in our research.

### Sociodemographic variables and appraisal of job demands as challenges and hindrances

Age proved to be a positive predictor of the appraisal of job demands as challenges and at the same time a positive predictor of their appraisal as hindrances. In other words, the older the employee, the more he or she assessed job demands as challenges, but also the more he or she assessed them as hindrances. It can be stated that age was conducive to the assessment of demands in a more explicit way. With age, moderate appraisals decreased, and more extreme appraisals, both positive and negative, intensified. Perhaps this result was determined by generational differences in that the sphere of work is more important for the older generations than for the younger ones, for whom the sphere of private life is relatively more important [76]. Moreover, what is more important is seen in more distinct colours.

Gender was not of much importance in the global appraisal of job demands as challenges and hindrances. When analysing individual types of job demands, it was found that only one type of job demands, work pace, was perceived by men as more challenging than women. Differences in the social roles of women and men could explain such a result. Although social roles are more and more equal, the need to combine professional and family responsibilities are more ascribed to women than to men. Therefore, time pressure at work is not perceived as a challenge by women as by men.

The respondents’ occupation has proved to be a particularly important predictor of appraisal of job demands as challenges. The IT employees assessed the demands of work as a challenge significantly higher than the two other occupational groups’ employees, i.e. health care and public transport. However, the type of industry did not affect the way the demands were appraised as hindrances. There are two possible explanations for the differences between industries in appraising job demands as challenges. Firstly, some occupations, especially those related to white-collar work, have better access to resources [77]. Thus, they are more predisposed to perceive job demands as challenges. This would explain why IT employees were characterised by a higher appraisal of job demands as challenges. This explanation is partially confirmed by the results of the regression analysis of demands’ appraisal as challenges. After entering psychosocial job resources and psychological capital to the regression model, the regression coefficient of the relationship between the occupational group and global challenge appraisal decreased significantly. In other words, some of the differences between occupation resulted from the differences in resources. The second possible explanation is that the occupations differ in their job demands structure. For example, cognitive demands are more specific to the IT employees and emotional demands to the healthcare employees. Thus, the type of prevailing demands could also determine the differences observed between the industries. However, as the ANOVA analysis proved, IT employees made a significantly higher appraisal of job demands as challenges in the case of every type of job demands (also emotional job demands, less intense in this group).

### Types of job demands and global appraisal of job demands as challenges and hindrances

When analysing the relationship between psychosocial job resources and psychological capital and the global appraisal of job demands as challenges and hindrances, the level of different types of demands was also controlled for.

Two types of demands, i.e. cognitive demands and work pace, played a significant role on global appraisal of job demands as challenges and hindrances. The level of cognitive demands was the predictor of global appraisal of job demands as challenges. This confirms the results of previous research on assessing cognitive demands mainly as challenges [3] and such a result provides a partial rationale for dividing demands into challenges and hindrances by researchers. It appears that while each type of job demands can be appraised both as a challenge and a hindrance, some types of demands, in our study, cognitive demands, are more related to challenge appraisal.

In turn, the work pace level has been a predictor of the global appraisal of job demands as both challenges and hindrances. This result confirms the twofold perception of time pressure already presented by Searle and Auton [10] and Chong, Van Eerde, Chai, and Rutte [6] which may lead to different psychological consequences [78].

Based on the results, it cannot be ruled out that the overall appraisal of job demands as challenges and/or hindrances is influenced not only by the type of job demands, but also by various prevalence and perception of particular demands across different occupations. The fact that challenge appraisal for all types of job demands was significantly higher in IT employees, may be the reason why emotional demands, amongst other job demands, were not related to the global appraisal of job demands, either as challenges or hindrances. Working in IT tends to be associated with lower levels of emotional demands than e.g. working with patients [79, 80]. It shows why the level of job demands is not always a sufficient indicator for their appraisal. Further research could be conducted in order to find other factors that would explain differences in the relationship between various types of job demands and their appraisal.

It is also worth noting that the result on emotional demands indicating that they are appraised as challenges to a higher degree than hindrances contradicts the approach of some researchers who treated emotional demands as hindrances [3], but is similar to results obtained by Bakker and Sanz-Vergel [60].

In general, the research has shown that the type of demands have a role to play in explaining the global appraisal of work demands in terms of hindrance and challenge. Nevertheless, it has also been shown that even when controlling for the type of job demands, the psychosocial and individual resources of the employee played a significant role in the appraisal.

## Limitations and future directions

The study has some limitations. First of all, the study was conducted in a cross-sectional design, making it impossible to interpret the results in cause-and-effect relationships. Therefore, it cannot be concluded whether people with high individual and job resources perceive job demands more as challenges due to these resources or are simply more likely to make positive appraisals of the environment. These doubts could be resolved in a longitudinal study. Moreover, the results were obtained using multiple imputation technique, as a way of dealing with missing data in the database. From the theoretical point of view, multiple imputation is the optimal method when handling missing data [81], in comparison to listwise deletion and single imputation [82, 83]. However, it should be noted that missing data should be prevented as it may reduce the statistical power of a study [82].

Secondly, the chosen study group could be both a strength and a limitation. Three occupational groups could be not enough to draw conclusions on the studied relationships in the general working populations, especially considering various working conditions, including job demands and resources, which seem to play a role in the individual appraisal of job demands. On the other hand, three occupational groups included in our study represent sectors characterised by a high level of job demands and work intensity [80], with differences in particular job demands levels. By including these groups, it was possible to grasp different sectors’ role in the job demands appraisals. As was presented in Bakker and Sanz-Vergel [60], analysing job demands as challenges or hindrances according to occupational group appraisal, explains unexpected relationships between emotional demands or work pressure and wellbeing. However, including more occupational groups in future studies could allow for presenting more general relationships.

Moreover, the disproportion of gender distribution across three occupational groups also should be considered when interpreting the results, because of differences in job demands and resources. Because of such disproportion, we were not able to perform analyses taking into account the combined effects of occupation and gender. It would be interesting to see future studies avoiding such disproportion even if the study group would not reflect the actual gender distribution in a given population.

The measurement of the appraisal of job demands as challenges and hindrances can create some difficulties in terms of interpretation of the results. As the appraisal was conducted on two separate scales, high scores on one scale may have accompanied high scores on the other, making it difficult to draw conclusions. Moreover, the fact that an employee assessed a given type of job demands low on the scale of challenges may have meant that this requirement was not a significant stressor for the employee–rather than that he or she perceived the demand less positively. Therefore, further studies could also analyse the level of stress associated with the demand [84] or combine the level of job demands with their appraisal in terms of challenges or hindrances. Cavanaugh at el. [1] on the other hand, investigated the level of stress associated with the demands and hindrances, which in turn did not fill the gap related to the lack of cognitive appraisal of individual workers and the nature of the demand.

## Conclusions

The study results show that psychosocial job resources (horizontal and vertical trust) were more strongly related with the subjective appraisal of job demands than individual resources. Age, occupation and some job demands also played an important role. More significant relationships were found when analysing predictors of the challenge appraisal than hindrance appraisal of job demands. The take home message for workplaces is as follows: interventions directed at an increasing mutual trust between employees as well as increasing psychological capital may lead to positive outcomes. Not only because they allow us to develop the ability to cope with job demands. They also lead to positive effects since they could influence a cognitive appraisal of these demands in terms of challenges. Such a perception—as proved in other studies—promotes employee wellbeing and productivity.

## Supporting information

S1 Data (SAV)

## References

[1] Cavanaugh M, Boswell W, Roehling M, Boudreau J. An empirical examination of self-reported work stress among U.S. managers. J Appl Psychol. 2000;85(1):65–74. 10.1037/0021-9010.85.1.65 10740957

[2] Tadić M, Bakker AB, Oerlemans WGM. Challenge versus hindrance job demands and well-being: A diary study on the moderating role of job resources. J Occup Organ Psychol. 2015; 88:702–725.

[3] van den Broeck A, De Cuyper N, De Witte H, Vansteenkiste M. Not all job demands are equal: Differentiating job hindrances and job challenges in the Job Demands-Resources model. Eur J Work Organ Psy. 2010;19(6):735–759.

[4] Lepine J, Podsakoff N, Lepine M. A Meta-Analytic Test of the Challenge Stressor-Hindrance Stressor Framework: An Explanation for Inconsistent Relationships among Stressors and Performance. Acad Manage J. 2005;48(5):764–775.

[5] Podsakoff NP, Lepine JA, Lepine MA. Differential challenge stressor-hindrance stressor relationships with job attitudes, turnover intentions, turnover, and withdrawal behavior: A meta-analysis. J Appl Psychol. 2007;92(2):438–454. 10.1037/0021-9010.92.2.438 17371090

[6] Chong DS, Van Eerde W, Chai KH, Rutte CG. A double-edged sword: The effects of challenge and hindrance time pressure on new product development teams. IEEE Trans Eng Manag. 2010;58(1):71–86.

[7] Crawford ER, LePine JA, Rich BL. Linking job demands and resources to employee engagement and burnout: A theoretical extension and meta-analytic test. J Appl Psychol. 2010;95:834–848. 10.1037/a0019364 20836586

[8] Tuckey MR, Searle B, Boyd CM, Winefield AH, Winefield HR. Hindrances are not threats: Advancing the multidimensionality of work stress. J Occup Health Psychol. 2015 4;20(2):131–47. 10.1037/a0038280 25365630

[9] Edwards BD, Franco-Watkins AM, Cullen KL, Howell JW, Acuff RE Jr. Unifying the challenge-hindrance and sociocognitive models of stress. Int J Stress Manag. 2014;21(2):162–185.

[10] Searle BJ, Auton JC. The merits of measuring challenge and hindrance appraisals. Anxiety Stress Coping. 2015;28:121–143. 10.1080/10615806.2014.931378 24901872

[11] Webster JA, Beehr TA, Love K. Extending the challenge-hindrance model of occupational stress: The role of appraisal. J Vocat Behav. 2011;79:505–516.

[12] Lazarus RS, Folkman S. Stress, appraisal and coping. New York: Springer; 1984.

[13] Tomaka J, Blascovich J, Kibler J, Ernst JM. Cognitive and physiological antecedents of threat and challenge appraisal. J Pers Soc Psychol. 1997 7;73(1):63–72. 10.1037//0022-3514.73.1.63 9216079

[14] Hitchcock C, Ellis AA, Williamson P, Nixon RD. The prospective role of cognitive appraisals and social support in predicting children’s posttraumatic stress. J Abnorm Child Psychol. 2015;43(8):1485–1492. 10.1007/s10802-015-0034-7 25971884 PMC4607721

[15] Karasek R, Theorell T. Healthy Work: Stress, Productivity, and the Reconstruction of Working Life. New York: Basic Books; 1990.

[16] Demerouti E, Bakker A, Nachreiner F, Schaufeli W. The Job Demands–Resources Model of Burnout. J Appl Psychol. 2001;86:499–512. 10.1037/0021-9010.86.3.499 11419809

[17] Fukuyama F. Trust: The social virtues and the creation of prosperity. New York: Free press; 1995.

[18] Putnam RD. Bowling alone: America’s declining social capital. J Democr. 1995;6:65–78.

[19] Hobfoll S. Conservation of Resources. A New attempt at conceptualizing stress. Am Psychol. 1989;44(3):513–524. 10.1037//0003-066x.44.3.513 2648906

[20] Xanthopoulou D, Bakker AB, Demerouti E, Schaufeli WB. The role of personal resources in the job demands-resources model. Int J Stress Manage. 2007;14(2):121–141.

[21] Schaufeli WB, Bakker AB. Job demands, job resources, and their relationship with burnout and engagement: A multi‐sample study. J Organ Behav. 2004;25(3):293–315.

[22] Bakker A, Hakanen J, Demerouti E, Xanthopoulou D. Job resources boost work engagement, particularly when job demands are high. J Educ Psychol. 2007;99(2):274–284.

[23] Lambert EG, Hogan NL, Barton-Bellessa SM, Jiang S. Examining the relationship between supervisor and management trust and job burnout among correctional staff. Crim Justice Behav. 2012;39(7):938–957.

[24] Karasek R, Brisson C, Kawakami N, Houtman I, Bongers P, Amick B. The Job Content Questionnaire (JCQ): an instrument for internationally comparative assessments of psychosocial job characteristics. J Occup Health Psychol. 1998:3(4);322–355. 10.1037//1076-8998.3.4.322 9805280

[25] Pejtersen JH, Kristensen TS, Borg V, Bjorner JB. The second version of the Copenhagen Psychosocial Questionnaire. Scand J Public Health. 2010;38(3_suppl):8–24. 10.1177/1403494809349858 21172767

[26] Dicke T, Marsh HW, Riley P, Parker PD, Guo J, Horwood M. Validating the Copenhagen Psychosocial Questionnaire (COPSOQ-II) using set-ESEM: Identifying psychosocial risk factors in a sample of school principals. Front Psychol. 2018;9:584. 10.3389/fpsyg.2018.00584 29760670 PMC5936966

[27] Freiman T, Merisalu E. Work-related psychosocial risk factors and mental health problems amongst nurses at a university hospital in Estonia: a cross-sectional study. Scand J Public Health. 2015 7;43(5):447–52. 10.1177/1403494815579477 25851017

[28] Dupret É, Bocéréan C, Teherani M, Feltrin M. The COPSOQ: a new French questionnaire for psychosocial risk assessment. Sante Publique. 2012;24(3):189–207. 23043694

[29] Andersen L, Fishwick D, Robinson E, Wiezer N, Mockałło Z, Grosjean V. Job satisfaction is more than a fruit basket, health checks and free exercise: Cross-sectional study among 10,000 wage earners. Scand J Public Health. 2017;45(5):476–484. 10.1177/1403494817698891 28381123 PMC5644715

[30] Nützi M, Koch P, Baur H, Elfering A. Work–Family conflict, task interruptions, and influence at work predict musculoskeletal pain in operating room nurses. Saf Health Work. 2015 12;6(4):329–37. 10.1016/j.shaw.2015.07.011 26929846 PMC4682021

[31] Sundstrup E, Andersen LL. Joint association of physical and psychosocial working conditions with risk of long-term sickness absence: Prospective cohort study with register follow-up. Scand J Public Health. 2020 6 29;1403494820936423. 10.1177/1403494820936423 32597327

[32] Clausen T, Burr H, Borg V. Do psychosocial work conditions predict risk of disability pensioning? An analysis of register-based outcomes using pooled data on 40,554 observations. Scand J Public Health. 2014 6;42(4):377–84. 10.1177/1403494814527187 24637676

[33] Sewdas R, Thorsen SV, Boot CRL, Bjørner JB, Van der Beek AJ. Determinants of voluntary early retirement for older workers with and without chronic diseases: A Danish prospective study. Scand J Public Health. 2020 3;48(2):190–199. 10.1177/1403494819852787 31319774 PMC7042495

[34] Widerszal-Bazyl M. Kopenhaski Kwestionariusz Psychospołeczny (COPSOQ)–właściwości psychometryczne wybranych skal w polskiej wersji (Copenhagen Psychosocial Questionnaire (COPSOQ)—psychometric properties of selected scales in the Polish version). Med Pr. 2017;68(3):329–348. 10.13075/mp.5893.00443 28512362

[35] Kaiseler M, Polman RC, Nicholls AR. Gender differences in appraisal and coping: an examination of the situational and dispositional hypothesis. Int J Sport Psychol 2012;43(1):1–14.

[36] Nuebling M, Seidler A, Garthus-Niegel S, Latza U, Wagner M, Hegewald J. The Gutenberg Health Study: measuring psychosocial factors at work and predicting health and work-related outcomes with the ERI and the COPSOQ questionnaire. BMC Public Health. 2013 6 4;13:538. 10.1186/1471-2458-13-538 23734632 PMC3707767

[37] Moncada S, Utzet M, Molinero E, Llorens C, Moreno N, Galtés A, et al. The Copenhagen Psychosocial Questionnaire II (COPSOQ II) in Spain—a tool for psychosocial risk assessment at the workplace. Am J Ind Med. 2014 1;57(1):97–107. 10.1002/ajim.22238 24009215

[38] Bjorner J, Pejtersen J. Evaluating construct validity of the second version of the Copenhagen Psychosocial Questionnaire through analysis of differential item functioning and differential item effect. Scand J Public Health. 2009;38(3_suppl):90–105. 10.1177/1403494809352533 21172775

[39] Ashleigh M, Higgs M, Dulewicz V. A new propensity to trust scale and its relationship with individual well-being: implications for HRM policies and practices. Hum Resour Manag J. 2012;22(4):360–376.

[40] Helliwell JF, Huang H. Well-Being and Trust in the Workplace. J Happiness Stud. 2011;12:747–767.

[41] Berthelsen H, Pejtersen J, Söderfeldt B. Measurement of social support, community and trust in dentistry. Community Dent Oral Epidemiol. 2010;39(4):289–299. 10.1111/j.1600-0528.2010.00593.x 21091526

[42] Fink M, Kessler A. Cooperation, trust and performance–empirical results from three countries. British Journal of Management, 21(2), 469–483.

[43] Brown S, Gray D, McHardy J, Taylor K. Employee trust and workplace performance. J Econ Behav Organ. 2015;116:361–378.

[44] Burr H, Berthelsen H, Moncada S, Nübling M, Dupret E, Demiral Y, et al. The Third Version of the Copenhagen Psychosocial Questionnaire. Saf Health Work. 2019;10(4):482–503. 10.1016/j.shaw.2019.10.002 31890332 PMC6933167

[45] Gerich J. (2014). Effects of social networks on health from a stress theoretical perspective. Soc Indic Res. 2010;118(1):349–364.

[46] Wind TR, Komproe IC. The mechanisms that associate community social capital with post-disaster mental health: a multilevel model. Soc Sci Med. 2012;75(9):1715–1720. 10.1016/j.socscimed.2012.06.032 22883254

[47] Muurinen C, Laine M, Pentti J, Virtanen M, Salo P, Kivimäki M, et al. Vertical and horizontal trust at work as predictors of retirement intentions: the Finnish Public Sector Study. PLoS One. 2014 9 5;9(9):e106956. 10.1371/journal.pone.0106956 25191745 PMC4156392

[48] Luthans F, Avolio B, Avey J, Norman S. Positive Psychological Capital: Measurement and Relationship with Performance and Satisfaction. Pers Psychol. 2007;60:541–572.

[49] Choi Y, Lee D. Psychological capital, big five traits, and employee outcomes. J Manage Psychol. 2014;29(2):122–140.

[50] Avey J, Reichard R, Luthans F, Mhatre K. Meta-analysis of the impact of positive psychological capital on employee attitudes, behaviors, and performance. Hum Resour Dev Q. 2011;22(2):127–152.

[51] Jerusalem M, Schwarzer R. Self-efficacy as a resource factor in stress appraisal processes. In; Schwarzer R, editor. Self-efficacy: Thought control of action. Hemisphere Publishing Corp; 1992. pp. 195–213.

[52] Cash M, Gardner D. Cognitive hardiness, appraisal and coping: comparing two transactional models. J Manage Psychol. 2011;26(8):646–664.

[53] Science Ethics Committee. Code of ethics for research workers. Warsaw: Polish Academy of Sciences; 2017. https://instytucja.pan.pl/index.php/kodeks-etyki-pracownika-naukowego

[54] European Commission. Ethics in Social Science and Humanities; 2018. https://ec.europa.eu/research/participants/data/ref/h2020/other/hi/h2020_ethics-soc-science-humanities_en.pdf

[55] Whicher D, Wu AW. Ethics Review of Survey Research: A Mandatory Requirement for Publication? Patient. 2015;8(6):477–482. 10.1007/s40271-015-0141-0 26392006

[56] George D, Mallery P. SPSS for Windows step by step: A simple guide and reference 11.0 update (4th ed.). Boston: Allyn & Bacon; 2003.

[57] Nunnally JC. Psychometric theory (2nd ed.). New York: McGraw-Hill; 1978.

[58] Baka Ł. Kopenhaski Kwestionariusz Psychospołeczny (COPSOQ II). Podręcznik do polskiej wersji narzędzia (Copenhagen Psychosocial Questionnaire (COPSOQ II). The manual to the Polish version). Warsaw: CIOP-PIB; 2019.

[59] Luthans F, Youssef CM, Avolio BJ. Psychological capital: Developing the human competitive edge. Oxford: Oxford University Press; 2007.

[60] Bakker A, Sanz-Vergel A. Weekly work engagement and flourishing: The role of hindrance and challenge job demands. J Vocat Behav. 2013;83(3):397–409.

[61] Allik M, Kearns A. “There goes the fear”: feelings of safety at home and in the neighborhood: The role of personal, social, and service factors. J Community Psychol. 2016;45(4):543–63.

[62] Martínez L M, Estrada D, Prada SI. Mental health, interpersonal trust and subjective well-being in a high violence context. SSM Popul Health. 2019;8:100423. 10.1016/j.ssmph.2019.100423 31321278 PMC6612929

[63] Diener E, Suh EM. National differences in subjective well-being. In: Kahneman D, Diener E, Schwarz N, editors. Well-being: The foundations of hedonic psychology. Russell Sage Foundation; 1999. pp. 434–450.

[64] Barefoot J, Maynard K, Beckham J, Brummett B, Hooker K, Siegler I. Trust, health, and longevity. J Behav Med. 1998;21(6):517–526. 10.1023/a:1018792528008 9891252

[65] Hyyppä MT. Healthy ties: Social capital, population health and survival. Springer Science & Business Media; 2010.

[66] Tanghe J, Wisse B, van der Flier H. The role of group member affect in the relationship between trust and cooperation. Br J Manag. 2010;21(2):359–374.

[67] McAllister DJ. Affect-and cognition-based trust as foundations for interpersonal cooperation in organizations. Acad Manage J. 1995;38(1):24–59.

[68] Bauer P, Keusch F, Kreuter F. Trust and cooperative behavior: Evidence from the realm of data-sharing. PLoS One. 2019;14(8):e0220115. 10.1371/journal.pone.0220115 31433802 PMC6703688

[69] Eek D, Rothstein B. Exploring a Causal Relationship Between Vertical and Horizontal Trust. Quality of Government Working Paper Series. 2005;vol. 4. Göteborg University, Sweden. https://gupea.ub.gu.se/handle/2077/39200

[70] Thomsen M, Karsten S, Oort FJ. Social exchange in Dutch schools for vocational education and training: The role of teachers’ trust in colleagues, the supervisor and higher management. Educ Manag Adm Lead. 2015;43(5):755–771.

[71] Renzl B, Matzler K, Mader C. Impact of trust in colleagues and management on knowledge sharing within and across work groups. In: CD-Proceedings of the 6th European Conference on Organizational Knowledge, Learning, and Capabilities. Bentley College, Boston; 2005.

[72] Mattila M, Salminen H, Astahova A Coping with a boundaryless career–A focus on Finnish self-initiated expatriates in Poland. In: Habti D, Elo M, editors. Global Mobility of Highly Skilled People. Springer, Cham; 2019. pp. 207–229.

[73] Peeters M, Buunk B, Schaufeli W. Social Interactions, Stressful Events And Negative Affect At Work—A Microanalytic Approach. Eur J Soc Psychol. 1995;25(4):391–401.

[74] De Jonge J, Dormann Ch. The DISC Model: Demand-Induced Strain Compensation mechanisms in job stress. In Dollard MF, Winefield AH, Winefield HR, editors. Occupational Stress in the Service Professions. London: Taylor & Francis; 2003. pp. 43–74.

[75] Min H, Kim HJ, Lee SB. Extending the challenge-hindrance stressor framework: the roleof psychological capital. Int J Hosp Manag. 2015;50:105–114.

[76] Twenge JM, Campbell SM, Hoffman BJ, Lance CE. Generational differences in work values: Leisure and extrinsic values increasing, social and intrinsic values decreasing. J Manage. 2010;36(5):1117–1142.

[77] Raittila S, Rahkonen O, Lahelma E, Alho J, Kouvonen A. Occupational class differences in trajectories of working conditions in women. Int J Environ Res Public Health. 2017 7;14(7):790. 10.3390/ijerph14060625 PMC5486311 28598380

[78] Widmer PS, Semmer NK, Kälin W, Jacobshagen N, Meier LL. The ambivalence of challenge stressors: Time pressure associated with both negative and positive well-being. J Vocat Behav. 2012;80(2):422–433.

[79] Nuebling M, Hasselhorn HM. The Copenhagen Psychosocial Questionnaire in Germany: from the validation of the instrument to the formation of a job-specific database of psychosocial factors at work. Scand J Public Health. 2010;38(3_suppl):120–124. 10.1177/1403494809353652 21172777

[80] Eurofound. Sixth European Working Conditions Survey–Overview report (2017 update). Luxembourg: Publications Office of the European Union; 2017.

[81] van Ginkel JR, Linting M, Rippe RC, van der Voort A. Rebutting existing misconceptions about multiple imputation as a method for handling missing data. J Pers Assess. 2020;102(3):297–308. 10.1080/00223891.2018.1530680 30657714

[82] Kang H. The prevention and handling of the missing data. Korean J Anesthesiol. 2013 5;64(5):402–406. 10.4097/kjae.2013.64.5.402 23741561 PMC3668100

[83] Van Buuren S. Flexible imputation of missing data. Chapman and Hall/CRC press; 2018.

[84] Siegrist J. Adverse health effects of high-effort/low-reward conditions. J Occup Health Psychol. 1996;1(1):27–41. 10.1037//1076-8998.1.1.27 9547031

